# Concurrent chemoradiotherapy with weekly docetaxel versus cisplatin in the treatment of locoregionally advanced nasopharyngeal carcinoma: a propensity score-matched analysis

**DOI:** 10.1186/s40880-019-0380-x

**Published:** 2019-06-27

**Authors:** Jun-Fang Liao, Qun Zhang, Xiao-Jing Du, Mei Lan, Shan Liu, Yun-Fei Xia, Xiu-Yu Cai, Wei Luo

**Affiliations:** 1 Department of Radiation Oncology, National Cancer Center/Cancer Hospital & Shenzhen Hospital, Shenzhen, 518116 Guangdong P. R. China; 2 Department of Radiation Oncology, The First Affiliated Hospital of Sun Yat-sen University, Guangzhou, 510080 Guangdong P. R. China; 3 State Key Laboratory of Oncology in South China, Collaborative Innovation Center for Cancer Medicine, Sun Yat-sen University Cancer Center, Guangzhou, 510060 Guangdong P. R. China; 4 Department of Radiation Oncology, State Key Laboratory of Oncology in South China, Collaborative Innovation Center for Cancer Medicine, Sun Yat-sen University Cancer Center, Guangzhou, 510060 Guangdong P. R. China; 5 Department of Radiation Oncology, Cancer Hospital Affiliated to School of Medicine, Chengdu, 610041 Sichuan P. R. China; 6 Department of VIP Region, Sun Yat-sen University Cancer Center, Guangzhou, 510060 Guangdong P. R. China

**Keywords:** Concurrent chemoradiotherapy, Docetaxel, Cisplatin, Nasopharyngeal carcinoma, Propensity score matching, Intensity-modulated radiotherapy, Overall survival, Distant metastasis-free survival, Locoregional recurrence-free survival, Nodal recurrence-free survival

## Abstract

**Background:**

Promising efficacy and manageable toxicity of docetaxel-based concurrent chemoradiotherapy (CCRT) were reported in head and neck cancer. In addition, the effect of CCRT in combination with cisplatin and/or 5-fluorouracil on both locoregionally advanced and metastatic/recurrent nasopharyngeal carcinoma (NPC) was verified. However, CCRT with docetaxel for locoregionally advanced NPC are not well studied. This study aimed to compare effectiveness and toxicities of CCRT with weekly docetaxel versus tri-weekly cisplatin for locoregionally advanced NPC.

**Methods:**

Clinical data of patients with locoregionally advanced NPC newly diagnosed between January 2010 and December 2014 receiving CCRT with either weekly docetaxel (15 mg/m^2^) or tri-weekly cisplatin (80–100 mg/m^2^) were reviewed. Propensity score matching at a 1:1 ratio was performed to balance baseline characteristics. Adverse events and survival were compared between the two groups.

**Results:**

A total of 962 patients were included as the whole cohort, and 448 patients were matched and were regarded as the matched cohort. The median follow-up duration was 48 months for the whole cohort. The 3-year nodal recurrence-free survival rate was significantly increased for patients treated with docetaxel in both the whole (hazard ratio [HR] = 0.37, 95% confidence interval [CI] 0.19–0.72, *P* = 0.030) and matched cohorts (HR = 0.33, 95% CI 0.14–0.79, *P* = 0.023). However, no significant differences were observed in overall survival, local recurrence-free survival, and distant metastasis-free survival between the two groups in both cohorts. Significantly higher rates of grade 3 radiodermatitis (6.7% vs. 1.8%, *P *= 0.001), mucositis (74.5% vs. 37.9%, *P* < 0.001), and leucopenia (2.2% vs. 11.6%, *P* < 0.001) were observed in the docetaxel group, but any grade of renal injury (1.8% vs. 15.1%, *P* < 0.001), vomiting (18.8% vs. 88.3%, *P* < 0.001), and ALT elevation (19.2% vs. 31.3%, *P* = 0.027) were more common in the cisplatin group.

**Conclusions:**

CCRT with weekly low-dose docetaxel is an effective and tolerable therapeutic regimen for locally advanced NPC. It provides a survival benefit mainly by improving the control of regional lymph node metastases, especially for patients with low pretreatment EBV DNA levels.

**Electronic supplementary material:**

The online version of this article (10.1186/s40880-019-0380-x) contains supplementary material, which is available to authorized users.

## Background

Nasopharyngeal carcinoma (NPC) is a fairly prevalent head and neck cancer in South China [1, 2]. Radiotherapy is the only radical treatment for NPC because of its anatomical limitations and hypersensitivity to radiotherapy [3]. Compared with two-dimensional conventional radiotherapy (2D-CRT), intensity-modulated radiotherapy (IMRT) has distinct advantages of better target coverage and risk-sparing of organs, and thus has been recognized as a more sophisticated treatment.

Benefits of cisplatin-based concurrent chemoradiotherapy (CCRT) for locally advanced NPC have been repeatedly verified by a clinical trial [4] and a meta-analysis [5] since the publication of the Intergroup 0099 trial [6]. Therefore, tri-weekly high-dose cisplatin together with radiotherapy has been currently considered the standard therapeutic strategy for locally advanced NPC. However, high-dose cisplatin is frequently associated with severe vomiting, ototoxicity, and renal dysfunction [7, 8], and only about 60% of patients were able to complete three planned treatment cycles in a clinical trial [9]. As such, the selection of patients with adequate renal function as candidates for high-dose cisplatin chemotherapy is important.

Docetaxel is a taxoid-class semisynthetic agent that promotes tubulin polymerization and the assembly of stable microtubules. In addition to cytotoxic activity, docetaxel has radiosensitizing activity which can arrest the cell cycle at the G_2_/M phase in highly radiosensitive cells [10, 11]. Promising activity and manageable toxicity of docetaxel-based CCRT were reported in breast cancer [12], non-small cell lung cancer [13], and head and neck cancer [14]. A previous meta-analysis reported that taxane-based CCRT had equivalent efficacy and toxicities to platinum-based regimens in head and neck squamous cell carcinoma [15]. In addition, its activity was verified in both locoregionally advanced and metastatic/recurrent NPC in combination with cisplatin [16] and/or 5-fluorouracil [17, 18]. However, studies on CCRT of IMRT with docetaxel for locoregionally advanced NPC are limited. Therefore, we conducted this propensity score matching analysis to compare the curative effect and toxicity between CCRT with weekly low-dose docetaxel and tri-weekly high-dose cisplatin for locoregionally advanced NPC in the IMRT era.

## Patients and methods

### Patient selection

This study was approved by the Ethics Committee of Sun Yat-sen University Cancer Center. We reviewed clinical records of patients with locoregionally advanced NPC diagnosed between January 2010 and December 2014. The inclusion criteria were as follows: (1) patients had biopsy-proven, histologically confirmed non-metastatic NPC based on the 7th edition of the American Joint Committee on Cancer (AJCC) staging system according to pretreatment magnetic resonance imaging (MRI) of the nasopharynx and neck, nasopharyngoscope, chest radiography or computed tomography (CT), abdominal sonography or CT, a whole-body bone scan or [18F]-fluorodeoxyglucose positron emission tomography/computed tomography (PET/CT); (2) patients underwent Epstein–Barr virus (EBV) DNA test prior to treatment; (3) patients received radical IMRT combined with weekly docetaxel (15 mg/m^2^) or tri-weekly cisplatin (80–100 mg/m^2^) concurrent chemotherapy. Plasma EBV DNA was quantified using quantitative real-time polymerase chain reaction (q-PCR) before treatment as previously described [19, 20]. The cutoff value of plasma EBV DNA level before the initiation of treatment was as previously established (4000 copies/mL) [21].

### IMRT

A detailed procedure of IMRT and target delineation has been reported previously [22]. The prescribed radiation dose to gross tumor volume of the nasopharynx (GTVnx) was 68–74 Gy, to gross tumor volume of lymph nodes (GTVnd) was 66–70 Gy, to high-risk clinical target volume (CTV1) was 60–66 Gy, and to low-risk clinical target volume (CTV2) was 50–56 Gy. All patients were treated once daily for 5 days a week, with a total of 30–33 fractions.

### Concurrent chemotherapy regimens

Patients were assigned to receive concurrent chemotherapy according to the National Comprehensive Cancer Network guidelines (Version 1.2018) [23] at the start of radiotherapy.

Docetaxel (Qilu Pharmacy Co., Ltd, Jinan, Shandong, China) was administered by drip infusion at the dose of 15 mg/m^2^ for 30 min once per week over 3 to 6 planned cycles. Dexamethasone (10 mg by intravenous infusion), cimetidine (200 mg by intravenous infusion), and diphenhydramine (40 mg by intramuscular injection) were given 30 min before docetaxel infusion for allergy prevention. After the completion of three cycles of docetaxel, chemotherapy was terminated if the nasopharyngeal and neck masses disappeared completely (assessed with electronic nasopharyngoscopy and physical examination) or severe mucosal toxicity led to weight loss exceeding 10%.

Cisplatin (Bristol-Myers Squibb Company, New York, USA) was administered every 3 weeks via intravenous infusion at the dose of 80–100 mg/m^2^ on days 1, 22, and 43 for 3 cycles concurrent with radiotherapy. The third cycle of chemotherapy was not given if radiotherapy was completed before the initiation of the third cycle once severe renal and hematologic adverse events occurred.

Pre-chemotherapy protective measures for cisplatin mainly included dexamethasone (10 mg by intravenous infusion), ondansetron (8 mg by intravenous infusion), and hydration.

### Adverse events and follow-up

Treatment-related adverse events were graded according to the National Cancer Institute Common Toxicity Criteria version 4.0 (CTCAE V4.0). The follow-up period was calculated from the date of diagnosis to either the latest follow-up or the date of death. Patients were examined every 3 months for the first 2 years and every 6 months thereafter. Nasopharyngoscopy, enhanced MRI of the head and neck, chest radiography, and abdominal ultrasound were routinely performed. The final date of follow-up was September 2017.

### Statistical analysis

All analyses were performed using SPSS version 22.0 (IBM Corporation, Armonk, NY, USA). The following endpoints were assessed: overall survival (OS), distant metastasis-free survival (DMFS), local recurrence-free survival (LRFS), and nodal recurrence-free survival (NRFS). Survival was calculated as the duration from the date of treatment to the date of death/first event or the last follow-up. The Kaplan–Meier method was used to estimate survival, and the differences were compared with the log-rank test. Cox proportional hazards model were used for multivariate analyses. Propensity score matching at a ratio of 1:1 was applied to create comparable cohorts of patients receiving concurrent chemotherapy with docetaxel or cisplatin. Covariates for matching included age, sex, histological type, pretreatment EBV DNA level, T stage, N stage, and clinical stage. The Chi-square test was used to test the balance of clinical characteristics and adverse events between the two groups. Two-sided *P* values less than 0.05 were considered significant.

## Results

### Patient characteristics

A total of 962 patients were selected for this study. The median numbers of concurrent cisplatin chemotherapy cycles in the whole and the matched cohorts were both 2 cycles (range 1–3 cycles). The median numbers of concurrent docetaxel chemotherapy cycles in the whole and matched cohorts were both 3 cycles (range 2–6 cycles). Table 1 shows the baseline characteristics and treatment details before and after the propensity score matching analysis. In the cisplatin group of the whole cohort, 8 (1.1%) patients received only 1 cycle of cisplatin: the concurrent chemotherapy was terminated for 5 patients because of acute coronary heart attack, grade 3 hepatic injury, repetitive hyponatremia, and renal injury, respectively; the second cycle of chemotherapy was changed to nedaplatin for 3 patients because of renal injury. In the docetaxel group, 1 (0.4%) patient received only 2 cycles of docetaxel due to the recurrence of a duodenal ulcer. Details of the completion of chemotherapy are shown in Additional file 1: Tables S1 and S2.

**Table 1:** Characteristics of the whole and propensity score-matched cohorts of patients with locoregionally advanced nasopharyngeal carcinoma

Characteristic	Whole cohort [cases (%)]				Propensity score-matched cohort [cases (%)]			
	Total	Cisplatin group	Docetaxel group	*P* value	Total	Cisplatin group	Docetaxel group	*P* value
Total	962	737	225		448	224	224	
Age				0.245				0.124
≤ 50 years	663 (68.9)	515 (69.9)	148 (65.8)		311 (69.4)	163 (72.8)	148 (66.1)	
> 50 years	299 (31.1)	222 (30.1)	77 (34.2)		137 (30.6)	61 (27.2)	76 (33.9)	
Sex				0.428				0.912
Male	716 (74.4)	544 (73.8)	172 (76.4)		341 (76.1)	170 (75.9)	171 (76.3)	
Female	246 (25.6)	193 (26.2)	53 (23.6)		107 (23.9)	54 (24.1)	53 (23.7)	
Histological type (WHO)								
I	4 (0.4)	3 (0.3)	1 (0.4)	0.939	2 (0.4)	1 (0.4)	1 (0.4)	1.000
II–III	958 (99.6)	734 (99.7)	224 (99.6)		446 (99.6)	223 (99.6)	223 (99.6)	
Clinical T stage^a^				0.337				0.278
T1–T2	226 (23.5)	171 (23.2)	55 (24.4)		114 (25.4)	59 (26.3)	55 (24.6)	
T3	591 (61.4)	461 (62.6)	130 (57.8)		268 (59.9)	138 (61.6)	130 (58.0)	
T4	145 (15.1)	105 (14.2)	40 (17.8)		66 (14.7)	27 (12.1)	39 (17.4)	
Clinical N stage^a^				0.049				0.959
N0	91 (9.5)	72 (9.8)	19 (8.4)		40 (8.9)	21 (9.4)	19 (8.5)	
N1	524 (54.5)	398 (54.0)	126 (56.3)		252 (56.3)	126 (56.3)	126 (56.3)	
N2	297 (30.8)	221 (30.0)	76 (33.8)		151 (33.7)	75 (33.5)	76 (33.9)	
N3	50 (5.2)	46 (6.2)	4 (1.8)		5 (1.1)	2 (0.9)	3 (1.3)	
Clinical stage^a^				0.109				0.189
II	123 (12.8)	85 (11.5)	38 (16.9)		80 (17.9)	42 (10.2)	38 (17.0)	
III	645 (67.0)	501 (68.0)	144 (64.0)		298 (66.5)	154 (68.8)	144 (64.3)	
IV	194 (20.2)	151 (20.5)	43 (19.1)		70 (15.6)	28 (12.5)	42 (18.7)	
Pretreatment EBV DNA				0.258				0.694
< 4000 copies/mL	633 (65.8)	492 (66.8)	141 (62.7)		286 (63.8)	145 (64.7)	141 (62.9)	
≥ 4000 copies/mL	329 (34.2)	245 (33.2)	84 (37.3)		162 (36.2)	79 (35.3)	83 (37.1)	

*WHO* World Health Organization, *EBV* Epstein–Barr virus

^a^The 7th edition of the AJCC/UICC staging system was used for TNM classification

After propensity score matching analysis, 448 patients (224 pairs) were selected, and the clinical characteristics were well balanced between the docetaxel and cisplatin groups. Additional file 1: Table S3 summarizes nodal volume (the volume of positive lymph nodes) and dosimetric parameters of the two groups, and no differences were observed in the minimal dose, mean dose, maximal dose, and nodal volume.

### Treatment effects

**Fig. 1:**
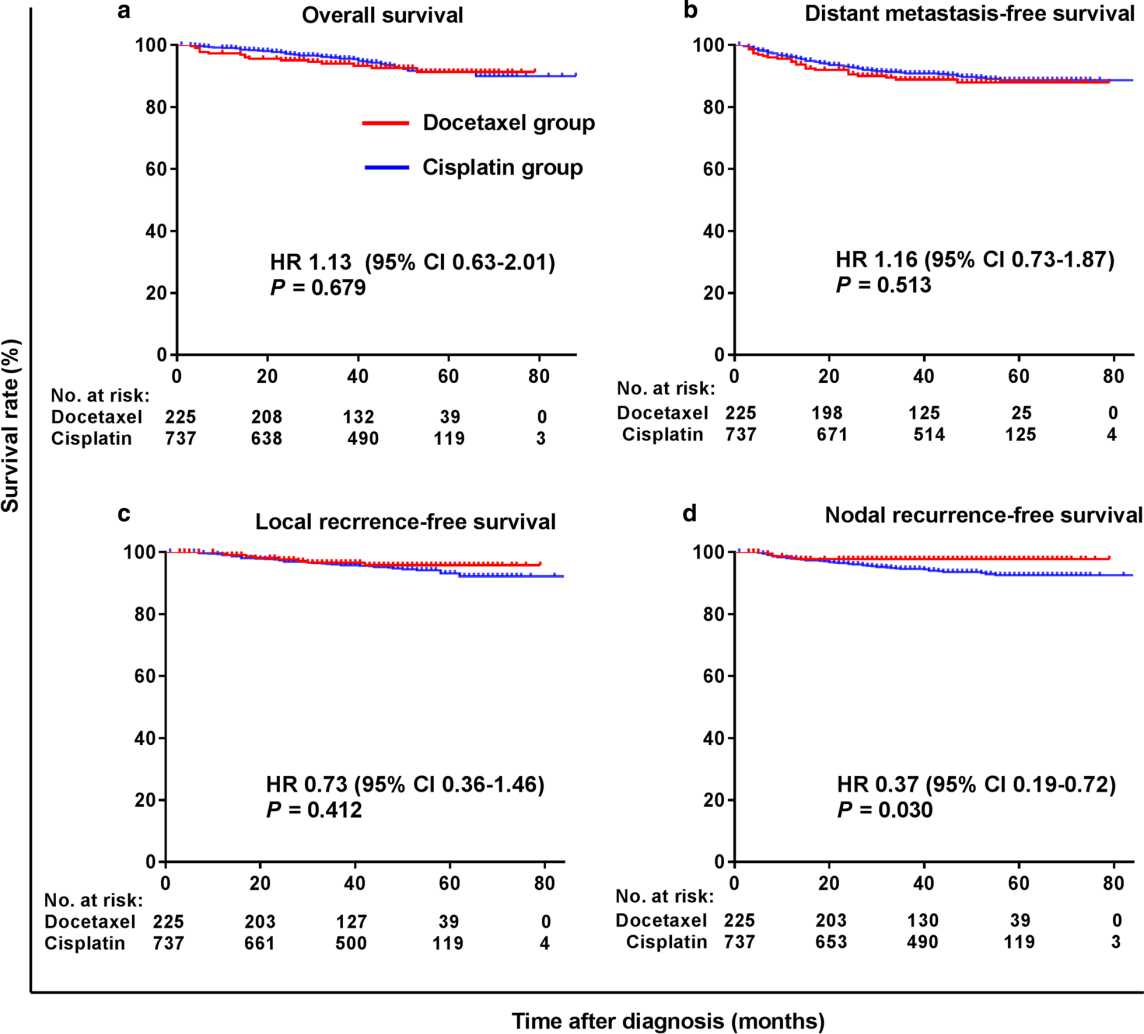
Kaplan–Meier survival curves of the 962 investigated patients in the whole cohort. **a** overall survival, **b** distant metastasis-free survival, **c** local recurrence-free survival, **d** Nodal recurrence-free survival

The median follow-up durations of the whole and matched cohorts were 48 months (range 1–88 months) and 47 months (range 3–89 months). Figure 1 shows the survival curves of the whole cohort. Patients in the docetaxel group had significantly higher 3-year NRFS rate than those in the cisplatin group (HR = 0.37, 95% CI 0.19–0.72, *P* = 0.030), whereas no significant differences in the 3-year OS, DMFS, and LRFS rates were observed between the two groups.

**Fig. 2:**
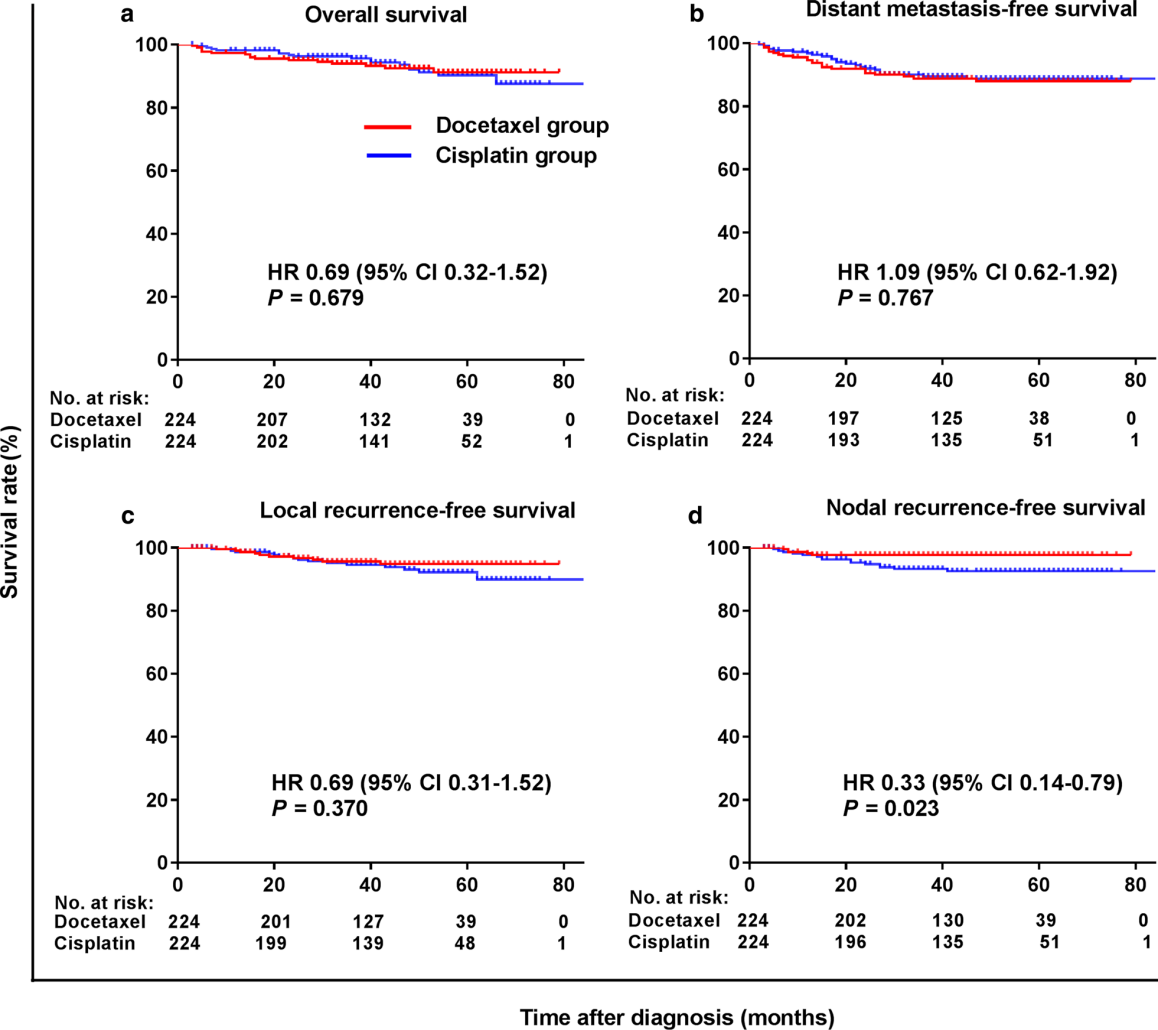
Kaplan–Meier survival curves of the 448 patients in the propensity score-matched cohort. **a** Overall survival; **b** distant metastasis-free survival; **c** Local recurrence-free survival; **d** Nodal recurrence-free survival

In the matched cohort, 9 (4.0%) patients experienced local recurrence, 5 (2.2%) developed regional recurrence, and 25 (11.2%) had distant metastasis in the docetaxel group; 13 (5.8%), 15 (6.7%), and 23 (10.2%) patients developed local recurrence, regional recurrence, and distant metastasis, respectively, in the cisplatin group. Survival curves for the matched cohort are shown in Fig. 2. Similarly, patients in the docetaxel group had significantly higher 3-year NRFS rate than those in the cisplatin group (HR = 0.33, 95% CI 0.14–0.79, *P* = 0.023), whereas the 3-year OS, DMFS, and LRFS rates did not differ significantly between the two groups.

In multivariate analysis (Table 2), concurrent docetaxel chemotherapy was the only independent prognostic factor for NRFS (HR = 0.34, 95% CI 0.12–0.93, *P* = 0.036), but not for OS, LRFS, and DMFS.

**Table 2:** Adjusted Cox multivariate analyses of prognostic factors for the matched cohort

Endpoint	Variable	*P* value	HR	95% CI
OS	Gender (male vs. female)	0.334	0.62	0.24–1.63
	Age (≤ 50 vs. > 50 years)	0.175	1.63	0.80–3.30
	Pretreatment EBV DNA (< 4000 vs. ≥ 4000 copies/mL)	0.766	1.12	0.54–2.29
	T stage (T1–2 vs. T3–4)	0.393	1.54	0.57–4.12
	N stage (N0–1 vs. N2–3)	0.022	2.26	1.12–4.53
	Clinical stage (II–III vs. IV)	0.134	1.86	0.83–4.18
	Concurrent chemotherapy (docetaxel vs. cisplatin)	0.802	0.92	0.46–1.83
DMFS	Gender (male vs. female)	0.885	0.95	0.48–1.88
	Age (≤ 50 vs. > 50 years)	0.901	0.96	0.52–1.79
	Pretreatment EBV DNA (< 4000 vs. ≥ 4000 copies/mL)	0.039	1.85	1.030–3.31
	T stage (T1–2 vs. T3–4)	0.600	1.22	0.59–2.51
	N stage (N0–1 vs. N2–3)	0.017	2.01	1.14–3.58
	Clinical stage (II–III vs. IV)	0.530	1.27	0.61–2.64
	Concurrent chemotherapy (docetaxel vs. cisplatin)	0.827	1.07	0.60–1.88
LRFS	Gender (male vs. female)	0.146	1.85	0.81–4.21
	Age (≤ 50 vs. ≥ 50 years)	0.986	0.99	0.41–2.42
	Pretreatment EBV DNA (< 4000 vs. ≥ 4000 copies/mL)	0.852	0.92	0.39–2.17
	T stage (T1–2 vs. T3–4)	0.418	1.58	0.52–4.79
	N stage (N0–1 vs. N2–3)	0.128	1.86	0.84–4.13
	Clinical stage (II–III vs. IV)	0.278	0.65	0.29–1.45
	Concurrent chemotherapy (docetaxel vs. cisplatin)	0.288	0.65	0.29–1.45
NRFS	Gender (male vs. female)	0.337	0.55	0.16–1.88
	Age (≤ 50 vs. > 50 years)	0.146	0.40	0.12–1.38
	Pretreatment EBV DNA (< 4000 vs. ≥ 4000 copies/mL)	0.171	1.86	0.77–4.52
	T stage (T1–2 vs. T3–4)	0.491	0.72	0.28–1.86
	N stage (N0–1 vs. N2–3)	0.334	1.55	0.64–3.77
	Clinical stage (II–III vs. IV)	0.783	0.81	0.18–3.71
	Concurrent chemotherapy (docetaxel vs. cisplatin)	0.036	0.34	0.12–0.93

*OS* overall survival, *EBV* Epstein–Barr virus, *DMFS* distant metastasis-free survival, *LRFS* locoregional recurrence-free survival, *NRFS* nodal recurrence-free survival, *HR* hazard ratio, *CI* confidence interval

### Subgroup analyses according to pretreatment EBV DNA levels for the matched cohort

**Fig. 3:**
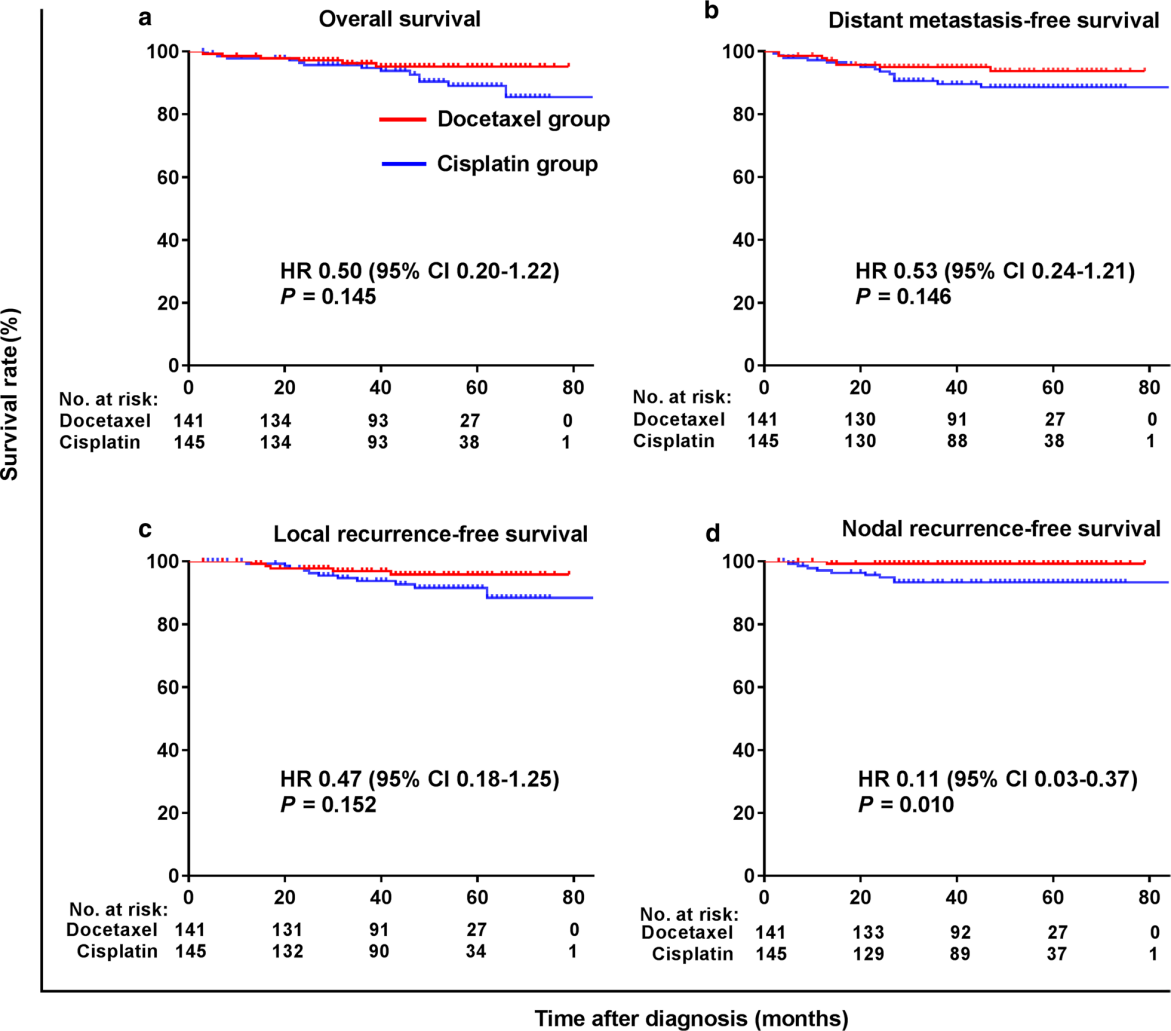
Kaplan–Meier survival curves of patients with pretreatment EBV DNA < 4000 copies/mL in the matched cohort. **a** Overall survival; **b** distant metastasis-free survival; **c** local recurrence-free survival; **d** Nodal recurrence-free survival

**Fig. 4:**
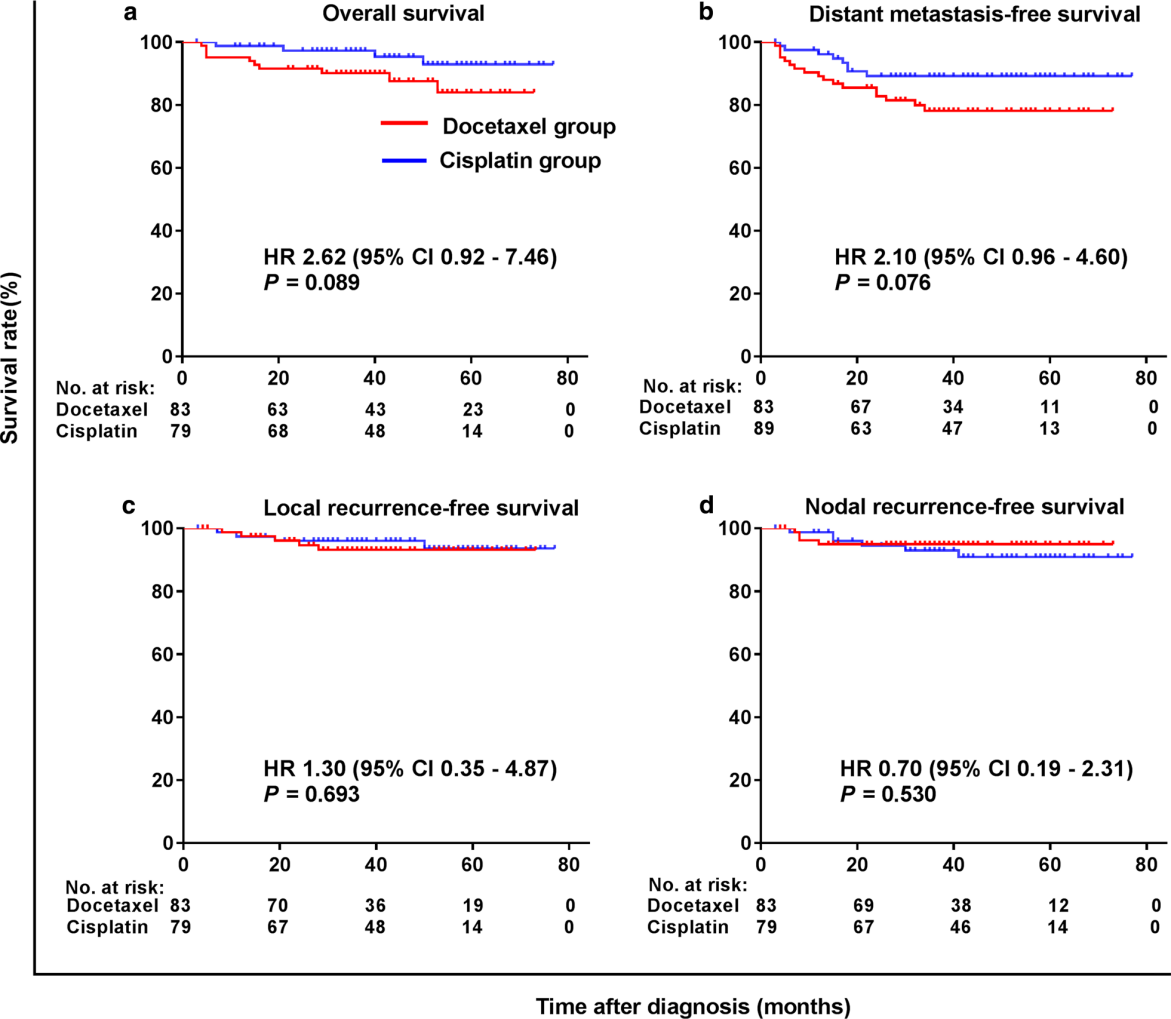
Kaplan–Meier survival curves of patients with pretreatment EBV DNA ≥ 4000 copies/mL in the matched cohort. **a** Overall survival; **b** distant metastasis-free survival; **c** local recurrence-free survival; **d** Nodal recurrence-free survival

Figures 3 and 4 illustrate the subgroup survival analyses according to pretreatment levels of EBV DNA in the matched cohort. Docetaxel was associated with a significant improvement in NRFS (HR = 0.11, 95% CI 0.03–0.37, *P* = 0.010) for patients with pretreatment EBV DNA level < 4000 copies/mL (Fig. 3).

For patients with a pretreatment EBV DNA level ≥ 4000 copies/mL, no significant differences in survival were observed between the two treatment groups (Fig. 4). This was possibly related to the small sample size that was underpowered to show statistical differences between two groups.

### Adverse events

Table 3 summarizes the chemoradiotherapy-related acute adverse events in the matched cohort. Higher rates of leucopenia, anemia, and thrombocytopenia were observed in the cisplatin group. In addition, acute renal injury, vomiting, and hepatic injury were also more common in the cisplatin group.

**Table 3:** Chemoradiotherapy-related acute adverse events in the matched cohort

Adverse event	Docetaxel group [cases (%)]		Cisplatin group [cases (%)]		*P* value
	Grade 1–2	Grade 3	Grade 1–2	Grade 3	
Leucopenia	90 (40.2)	5 (2.2)	164 (73.2)	26 (11.6)	< 0.001
Anemia	55 (24.6)	0	105 (46.8)	2 (0.9)	< 0.001
Thrombocytopenia	3 (1.3)	0	41 (18.3)	4 (1.8)	< 0.001
ALT elevation	42 (18.8)	1 (0.4)	68 (30.4)	2 (0.9)	0.027
CCR elevation	4 (1.8)	0	33 (14.7)	1 (0.4)	< 0.001
Vomiting^a^	42 (18.8)	0	185 (82.5)	13 (5.8)	< 0.001
Mucositis^a^	45 (20.1)	167 (74.5)	102 (45.5)	85 (37.9)	< 0.001
Weight loss^a^	171 (76.3)	0	166 (74.1)	2 (0.1)	0.010
Radiodermatitis^a^	191 (85.3)	15 (6.7)	185 (82.6)	4 (1.8)	0.001
Tinnitus^a^	23 (10.3)	0	24 (10.7)	0	0.835
Xerostomia^a^	171 (76.3)	2 (0.1)	170 (75.9)	2 (0.1)	0.052

Only one patient in the cisplatin group had grade 4 radiodermatitis

*ALT* Alanine transaminase, *AST* aspartate aminotransferase, *CCR* creatinine clearance

^a^Missing value: In the docetaxel group, medical records of 10 patients were missing for radiodermatitis, mucositis, and xerostomia, 8 for vomiting, 6 for weight loss, and 12 for tinnitus; In the cisplatin group, medical records of 35 patients were missing for radiodermatitis, 37 for mucositis, 38 for xerostomia, 9 for vomiting, 17 for weight loss, and 38 for tinnitus

Radiodermatitis and mucositis were the major adverse events observed in the docetaxel group. The rates of grade 3 radiodermatitis (6.7% vs. 1.8%, *P* = 0.001) and mucositis (74.5% vs. 37.9%, *P* < 0.001) were significantly higher in the docetaxel group than in the cisplatin group. Only one patient in the cisplatin group had grade 4 radiodermatitis. All acute adverse events were resolved with comprehensive care, and no treatment-associated deaths were observed in the two groups.

## Discussion

In the present study, we used a propensity score matching analysis to eliminate influences of confounding factors while assessing the curative effect and toxicity between CCRT with weekly low-dose docetaxel and tri-weekly high-dose cisplatin for locoregionally advanced NPC in the IMRT era. The results demonstrated that concurrent chemotherapy with docetaxel significantly increased the 3-year NRFS rate as compared with cisplatin (HR = 0.33, 95% CI 0.14–0.79, *P* = 0.03). However, the 3-year OS, DMFS, and LRFS rates were similar between the two groups.

Wei et al. [24] reviewed records of 73 stage III–IVA NPC patients to compare the efficacy of CCRT with docetaxel and cisplatin. Similar to our findings, they found no significant difference between the two groups in 3-year OS (86.5% vs. 92.5%, *P* = 0.298), DMFS (87.0% vs. 92.5%, *P* = 0.171), and local control rates (85.6% vs. 92.3%, *P* = 0.264). However, the distribution of their patient characteristics between the two treatment arms was unbalanced, and 2D-CRT was administered; whereas in the present study, all patients underwent IMRT, and propensity score matching was performed to balance baseline patient characteristics.

A previous study demonstrated that patients with pretreatment EBV DNA > 4000 copies/mL had a higher risk to develop distant metastasis compared with those with EBV DNA < 4000 copies/mL [21]. In the present study, it was interesting that docetaxel significantly increased 3-year NRFS rate as compared with cisplatin (99.3% vs. 93.3%, *P* = 0.010) for patients with pretreatment EBV DNA < 4000 copies/mL; although the OS, DMFS, and LRFS seemed longer in the docetaxel group, the differences were not significant. It is possible that concurrent weekly low-dose docetaxel mainly improved locoregional control due to its radiosensitization or synergistic effect with radiotherapy [25], but could not eradicate micro-metastases effectively for patients with a high risk of metastasis. This indicated that choosing concurrent chemotherapy regimens according to the pretreatment EBV DNA level might help to improve the curative effect.

The optimal dose and schedule of docetaxel remain poorly defined. The schedule of docetaxel consisted of mainly 6 cycles in previous studies [14, 24, 26, 27]. In the matched cohort of the present study, all patients in the docetaxel group received 3 cycles of chemotherapy, 54 (24.1%) received 4 or more cycles; 167 (74.6%) patients in the docetaxel group suffered grade 3 mucositis, which was much higher than those in previous studies (range from 27.9% to 61.4%) [14, 24, 28]. The main reason may be related to the use of IMRT which increases the radiation dose to the target area and thus leading to an increased risk of severe mucositis. Most patients completed the planned therapy, and none experienced grade 4 mucositis. Further research is needed to identify the optimal regimen of docetaxel to balance toxicity and curative effect in the IMRT era.

Previous studies revealed that mucositis was the most common restrictive factor in CCRT with docetaxel [27]. Several phase I/II studies reported that a weekly dose of docetaxel between 10 and 20 mg/m^2^ demonstrated acceptable toxicity and therapeutic activity [26, 28]. Calais et al. [27] reported a phase II trial of CCRT with weekly docetaxel at 20 mg/m^2^ for patients with stage III/IV oropharyngeal carcinoma. The rates of grade 3–4 mucositis (84%) and radiodermatitis (53%) were high, and grade 3–4 neutropenia was observed in 5% of patients. Jomon Raphael et al. [29] found that CCRT with weekly docetaxel at 15 mg/m^2^ for patients with locoregionally advanced, inoperable head and neck cancer was a feasible and suitable alternative to surgery, and the major adverse events were mucosal reaction (grade 3: 57%) and radiodermatitis (grade 3: 23%). Furthermore, the rates of grade 3 dysphagia and grade 2 weight loss (> 10% of the initial weight) were 38% and 23%. In the present study, CCRT with weekly docetaxel at 15 mg/m^2^ was well tolerated, and 224 (99.6%) patients had completed at least 3 courses concurrent chemotherapy. The rates of hematological adverse events, vomiting, liver impairment, and renal injury were lower in the docetaxel group than in the cisplatin group, whereas the rate of acute mucositis was higher in the docetaxel group.

The present study has several drawbacks inherent to observational analyses. For instance, the accuracy of recurrence evaluation or cause of death adjudication of observational analyses was inferior to that of a prospective clinical trial, which could result in possible misclassification of survival events. In addition, adverse reactions for some patients, especially for outpatients, were unrecorded.

## Conclusions

Our findings show that CCRT with weekly low-dose docetaxel is an effective and tolerable treatment for locally advanced NPC. It provides a survival benefit mainly by improving regional control, especially for patients with low pretreatment EBV DNA levels.

## Additional file

**Additional file 1: Table S1.** Details of the completion of chemotherapy in the whole cohort (962 patients) and the matched cohort (448 patients). **Table S2.** Details of the dose density of cisplatin in the whole cohort (737 patients) and the matched cohort (224 patients). **Table S3.** Nodal volume and dosimetric parameter of the two groups in the matched cohort.

## Data Availability

The datasets used or analyzed in the present study are available from the corresponding author on reasonable request. The key raw data have been deposited into the Research Data Deposit (http://www.researchdata.org.cn), with the approval number of RDDA2019001011.

## Abbreviations

CI: confidence interval
NPC: nasopharyngeal carcinoma
2D-CRT: two-dimensional conventional radiotherapy
IMRT: intensity modulated radiotherapy
CCRT: concurrent chemoradiotherapy
MRI: magnetic resonance imaging
q-PCR: quantitative real-time polymerase chain reaction
EBV: Epstein–Barr virus
CTCAE V4.0: Common Toxicity Criteria version 4.0
GTV: gross tumor volume
CTV: clinical tumor volume
OS: overall survival
DMFS: distant metastasis free survival
LRFS: local recurrence-free survival
NRFS: nodal recurrence-free survival
HR: hazard ratio
WHO: World Health Organization
NCCN: National Comprehensive Cancer Network
