# Correction to: Concurrent chemoradiotherapy with weekly docetaxel versus cisplatin in the treatment of locoregionally advanced nasopharyngeal carcinoma: a propensity score matched analysis

**DOI:** 10.1186/s40880-019-0396-2

**Published:** 2019-09-23

**Authors:** Jun-Fang Liao, Qun Zhang, Xiao-Jing Du, Mei Lan, Shan Liu, Yun-Fei Xia, Xiu-Yu Cai, Wei Luo

**Affiliations:** 1 Department of Radiation Oncology, National Cancer Center/National Clinical Research Center For Cancer/Cancer Hospital & Shenzhen Hospital, Chinese Academy of Medical Sciences and Peking Union Medical College, Shenzhen, 518116 China; 2 Department of Radiation Oncology, The First Affiliated Hospital of Sun Yat‑sen University, Guangzhou, 510080 Guangdong P. R. China; 3 State Key Laboratory of Oncology in South China, Collaborative Innovation Center for Cancer Medicine, Sun Yat‑sen University Cancer Center, Guangzhou, 510060 Guangdong P. R. China; 4 Department of Radiation Oncology, State Key Laboratory of Oncology in South China, Collaborative Innovation Center for Cancer Medicine, Sun Yat‑sen University Cancer Center, Guangzhou, 510060 Guangdong P. R. China; 5 Department of Radiation Oncology, Cancer Hospital Affiliated to School of Medicine, Chengdu, 610041 Sichuan P. R. China; 6 Department of VIP Region, Sun Yat‑sen University Cancer Center, Guangzhou, 510060 Guangdong, P. R. China

## Correction to: Cancer Commun (2019) 39:40 10.1186/s40880-019-0380-x

In the original publication of this article [1], the first affiliation name in the Author details section should be updated to ‘Department of Radiation Oncology, National Cancer Center/National Clinical Research Center for Cancer/Cancer Hospital & Shenzhen Hospital, Chinese Academy of Medical Sciences and Peking Union Medical College, Shenzhen 518116, China’ because the affiliation has been renamed from Jun. 15th, 2019.
