# Supplementary material for: Concurrent chemoradiotherapy with weekly docetaxel versus cisplatin in the treatment of locoregionally advanced nasopharyngeal carcinoma: a propensity score-matched analysis
Source: Cancer Commun (Lond). 2019 Jun 27;39:40. doi: 10.1186/s40880-019-0380-x (PMC6598276; doi:10.1186/s40880-019-0380-x)
Supplement: Supplementary file 1 — Additional file 1: Table S1. Details of the completion of chemotherapy in the whole cohort (962 patients) and the matched cohort (448 patients). Table S2. Details of the dose density of cisplatin in the whole cohort (737 patients) and the matched cohort (224 patients). Table S3. Nodal volume and dosimetric parameter of the two groups in the matched cohort. [file 40880_2019_380_MOESM1_ESM.docx]

Additional file 1

Table S1. Details of the completion of chemotherapy in the whole cohort (962 patients) and the matched cohort (448 patients).

| Chemotherapy cycle | Whole cohort [cases (%)] | | Propensity score-matched cohort [cases (%)] | |
| --- | --- | --- | --- | --- |
|  | Cisplatin group (*n* = 737) | Docetaxel group (*n* = 225) | Cisplatin group (*n* = 224) | Docetaxel group (*n* = 224) |
| 1 | 737 (100) | 225 (100) | 224 (100) | 224 (100) |
| 2 | 729 (98.9) | 225 (100) | 223 (99.6) | 224 (100) |
| 3 | 213 (31.3) | 224 (99.6) | 60 (26.8) | 223 (99.6) |
| 4 | 0 | 54 (24.0) | 0 | 53 (23.7) |
| 5 | 0 | 18 (8.0) | 0 | 18 (8.0) |
| 6 | 0 | 5 (2.2) | 0 | 5 (2.2) |

Table S2. Details of the dose density of cisplatin in the whole cohort (737 patients) and the matched cohort (224 patients).

| Dose density of cisplatin | Whole cohort [cases (%)] | Propensity score-matched cohort [cases (%)] |
| --- | --- | --- |
| <200 mg/m^2^ | 327 (44.4) | 114 (50.9) |
| ≥200 mg/m^2^ | 410 (55.6) | 110 (49.1) |

Table S3. Nodal volume and dosimetric parameter of the two groups in the matched cohort.

| Irradiation area | Dosimetric parameter | Docetaxel group | Cisplatin group | *P* value |
| --- | --- | --- | --- | --- |
| Left cervix | Nodal volume (mL) | 7.32 ± 9.48 | 8.89 ± 10.79 | 0.102 |
|  | Minimal dose (Gy) | 62.42 ± 3.21 | 62.84 ± 4.25 | 0.280 |
|  | Mean dose (Gy) | 68.20 ± 3.10 | 68.15 ± 3.43 | 0.884 |
|  | Maximal dose (Gy) | 71.98 ± 3.87 | 71.65 ± 3.67 | 0.390 |
| Right cervix | Nodal volume (mL) | 7.07 ± 10.23 | 7.76 ±8.81 | 0.504 |
|  | Minimal dose (Gy) | 61.92 ± 3.55 | 62.62 ± 3.70 | 0.061 |
|  | Mean dose (Gy) | 67.77 ± 3.25 | 67.95 ± 3.43 | 0.607 |
|  | Maximal dose (Gy) | 71.45 ± 3.73 | 71.38 ± 3.83 | 0.486 |
